# Survival of the wittiest (not friendliest): The art and science behind human linguistic and cognitive evolution

**DOI:** 10.1093/pnasnexus/pgag052

**Published:** 2026-03-31

**Authors:** Ljiljana Progovac

**Affiliations:** Linguistics Program, Wayne State University, 5057 Woodward, Detroit, MI 48202, USA

**Keywords:** language evolution, cognitive contest, quick-wittedness, humor, sexual selection

## Abstract

There are several previous findings, both theoretical and experimental, that have paved the way toward the view of human evolution as the “survival of the wittiest,” subject to sexual selection, offering better explanatory power than the “survival of the friendliest.” While research on language evolution has largely neglected the artistic dimension, the fitness in humans is correlated with linguistic eloquence, including humor. This is one of those cases where science can only explain a phenomenon by considering its artistic dimension, and in which linguistic creativity and genetics come into direct contact. My argument is that selection for quick-wittedness (using and combining words in a clever and funny way), specific to language and unique to humans, needs to be added to the complex picture of human evolution, relevant from the earliest stages of language/grammar. Wittiness is a trait that allows competition (by “outwitting” others) while at the same time favoring “friendliness” in that it provides an excellent platform for replacing physical aggression with verbal behavior and cognitive contest, the hallmarks of human nature. This proposal is based on a precise linguistic reconstruction of the earliest stages of grammar, providing linguistic detail (“living fossils”) necessary for hypothesis testing, including neuroimaging experiments.

“It could never have been anticipated that the power to charm the female has been in some few instances more important than the power to conquer the other males in battle.” (1)

## Language evolution

Much of linguistic research on language evolution has focused on human analytical abilities to form sentences with which to express (complex) propositions and thoughts. While this is certainly an important function of language(s) today, this is not the only function, and it cannot be expected to have been a major function in the earliest stages of language. Understanding the motivation behind evolving language at these earliest stages is at the heart of understanding how language took off, and how we humans as a species evolved. While some approaches deny the role of sexual selection in the evolution of language, or the possibility of gradual, adaptive evolution [e.g. (2–4)], several others have found it advantageous to invoke adaptation and sexual selection for specifically language skills [e.g. (5–10)]. Here, I show that some of these latter approaches have paved the way toward a scenario favoring the “survival of the wittiest,” in these earliest steps, but also beyond.

Darwin's (1) view was that language evolved gradually through sexual selection, and for him, language is “half art, half instinct,” but this creative dimension has largely been neglected in the present-day research on language evolution. Language evolution is one of the perhaps rare cases in which science can only explain a phenomenon by taking into account its artistic dimension. This article provides specific detail and analysis, which considers where and how the art and the science of human evolution intersect, allowing for hypothesis generation and testing.

But, first, what about the survival of the fittest, or the survival of the friendliest?

## Survival of the fittest? Or friendliest?

Charles Darwin's work [e.g. (1, 11, 12)] has been associated with the “survival of the fittest,” which is often (inaccurately) interpreted to be limited to the survival of the strongest, or the healthiest, physically speaking. But “fitness” in the biological sense is a much broader concept, in that it can refer to any trait which happens to provide a better survival rate in the (immediate) environment (natural or cultural), whatever that may be, such as the camouflage adaptations of many species, or increased lactose tolerance in societies that practice farming. Fitness in this sense certainly does not have to involve direct fighting or ruthlessness. It can be a slow, silent process over time, or it can be ratcheted up in some cases. Still, adaptation in the physical sense of fitness has certainly played an important role in the evolution of humans (and other species), and continues to do so, but see Evidence from genetics: What do grammar, taming of aggression, humor, and metaphoricity have in common? regarding possible effects of reduced physical aggression in humans. It is important to keep in mind that evolution can be about loss, and not just about gain.

As a seeming counterproposal to supposedly ruthless physical competition, “survival of the friendliest” has recently been proposed, that is, the view that *Homo sapiens* evolved via selection for prosociality, associated with a reduction in reactive aggression [e.g. (13, 14)]. It would appear at first sight that this is a more benevolent view of the human species, considering that it now does not seem to matter who is strong and ruthless, but rather who is friendly [see, for example, the interview with Brian Hare (15) in *The Washington Post*, implying (wrongly) that the two views are distinct]. However, in the scenario of the selection for “friendliness,” what one might consider as “friendly” types had to have competed (ruthlessly) against “unfriendly” types. In fact, as discussed in Wrangham (16), this would have included teaming up to kill alpha males. In the interview mentioned previously, Brian Hare is quoted as saying, “We are the friendliest human species that ever evolved, which has allowed us to outcompete other human species that are now extinct … When [the mechanism] is turned on, it allows us to win. We win by cooperation and teamwork.” For all our friendliness, we seem quite content with driving other species/populations to extinction, and we seem quite preoccupied with “winning” (this short quote has the word “win” in it twice).

There is no doubt that the ability to form alliances (to defeat common enemies) can be adaptive, but the term *friendliness* does not seem particularly suited for this trait. In this respect, Wrangham (16) refers to this trait as “groupishness,” characterizable as the ability to form groups and to cooperate within those groups, often in order to be better able to outcompete other groups or individuals. In this sense, the proposal of the survival of the friendliest (i.e. the most groupish) is not distinct from the original notion of the survival of the fittest, and it certainly does not do away with human ruthlessness. Groupishness has another dark side, the relatively recent rise of proactive (premeditated) aggression, as tracked in the archeological record [see, for example, (17, 18)]. According to Kissel and Kim (19), coordinated (groupish) violence, including widespread conflicts and wars associated with proactive aggression, became possible only recently, with the onset of symbolic thought and complex cognition. As such, it cannot be seen as a causal factor in the early entrenchment of language, but it can in fact be seen as facilitated by the very emergence and then complexification of language.

But, crucially, and most relevant for this article, these two aspects, selection for physical fitness, and selection for friendliness/groupishness, while both relevant for human evolution to some extent, do not begin to capture the essence of what it means to be human, specifically in relation to language. We may appreciate physical strength in our mates, and we may appreciate their friendly demeanor, but these are not necessarily traits that have to do with language directly, nor are these traits unique to humans [regarding the latter, consider for example the friendly demeanor in bonobos, as discussed in Hare (13) and Hare et al. (20)]. There is thus a need to supplement these general approaches to human evolution with the ones that directly and causally implicate human language and address human uniqueness. As pointed out previously, groupishness characterizes later stages in human evolution, and not the earliest stages, when language just started to emerge. The following sections address the crucial questions of how and why language took off in the first place [some discussion of the timing can be found in, for example, Progovac (10) and Progovac and Benítez-Burraco (21)].

As will be shown in this article, the view of human evolution as the “survival of the wittiest” offers more explanatory power and a more comprehensive approach than does the “survival of the friendliest.” This approach does cross-fertilize with the human self-domestication (HSD) hypothesis, via a feedback loop, thus also engaging the “friendliness” dimension (evidence from genetics: what do grammar, taming of aggression, humor, and metaphoricity have in common?). As such, it takes into account both competition and cooperation, avoiding unidimensional views of humans and their evolution. It gives an active, causal role to language in human evolution. It identifies the earliest stages of language, as well as certain “living fossils” of these stages, providing linguistic detail necessary for hypothesis testing.

## Survival of the wittiest (not friendliest): The role of sexual selection

“It can be said not that the chief is a man who speaks, but that he who speaks is a chief.” (22)

My argument is that selection for quick-wittedness and eloquence, specifically demonstrated by language and unique to humans, was relevant from the earliest stages of language evolution. Being witty and funny is certainly a rather desirable trait in humans even today, a form of art that we are not all equally good at, even though we all may strive to be. Individual variability in this respect also renders this trait subject to selection [see, for example, Vrticka et al. (23) for humor]. Wit and wittiness are characterizable as showing quick and inventive verbal humor, using words in a clever and funny way (Cambridge Dictionary; Merriam-Webster Dictionary) (see Evidence for sexual selection from colorful “fossils” for continuity of wittiness and nonverbal humor, found also in other species). Wittiness specifically refers to one's agility with words, including the ability to outwit others (i.e. to outcompete them with words). The most eloquent speakers tend to have the highest status, which in turn is correlated with greater reproductive success (24). Competition with language continues to this day, whether in subtle ways, or through outright verbal dueling attested across cultures around the world (8, 25).

This seems especially clear that traditional, oral societies across the globe, in which individuals who are unusually able to manipulate vocal, articulatory, and linguistic material, and thereby command attention, tend to enjoy higher than normal status and preferential access to positions of political power (25 , 26). In Amazonia the Pa’ikwené people are considered “good speakers” based on their “grammatical, rhetorical, and performative competence and vocal quality” (27). In the Trobriand Islands of New Guinea, lexical “power” seems to be achieved through the use of “archaisms, mythical names and strange compounds, formed according to unusual linguistic rules” (28). In New Zealand, “oratory is the prime qualification for entry into the power game” among the Maori (29). The Amuesha people in Central Peru describe a true leader as “the one who is powerful due to his or her words” (30). In Ethiopia among the Mursi, “the most frequently mentioned attribute of an influential man is his ability to speak well in public” (31). In Northern Transvaal, among the Venda people, “the greatest honour seems to be accorded to those who can manipulate words and sentences” (32). In South Africa, the Tshidi people consider oratorical ability as “a significant component of political success and the means by which politicians demonstrate their acumen” (33). In South America, “speaking is more than a privilege, it is a duty of the chief. It is to him that the mastery of words falls…It can be said not that the chief is a man who speaks, but that he who speaks is a chief” (22). According to Locke (8), this talent for oratory involves honest signaling, as it is “impossible to pretend a better knowledge of language than one really has, and to fake unusual skills in the delivery of speech. There are no individuals who seem eloquent, but in reality are not.”

Humans can, at least to a considerable extent, be characterized as smart and friendly, and yet, this cannot be the whole picture. We elect politicians to govern entire countries based on how witty and skillful they are at debates, and not based on how strong or friendly they are, or indeed how good they are at solving problems. We admire and replay the memorable snippets of witty exchanges at those debates, which are essentially verbal duels. Notably, we tend to fall for brief catchy phrases and nicknames, including the derogatory ones, rather than for sophisticated analyses of character/situation rendered in complex sentences or in statistical analysis. Rightly or wrongly, we often consider eloquence to be correlated with intelligence, but regrettably perhaps, being witty cannot be equated with being intelligent or wise, in the sense of making the best decisions, or solving problems in an optimal way. This is consistent with wittiness being associated with the artistic dimension of language.

The “survival of the wittiest” proposal is not an alternative to Darwin's notion of the survival of the fittest, but rather just a specific rendering of this approach when applied specifically to linguistic and cognitive evolution of humans. The argument I pursue in this article is that, since the emergence of language, and to this day, the fitness in humans has been highly correlated with their linguistic eloquence and agility, including clever and humorous uses of language. The following sections provide specific details and scenarios, including some results of experimental testing.

## Evidence from linguistic theories: Precise reconstruction of the earliest grammars

My discussion of the earliest grammars in language evolution, as well as of linguistic “fossils,” is firmly grounded in linguistic theory. In fact, while the “fossils” discussed in the following sections have turned out to be rather colorful and biologically relevant, identifying these “fossils” was made possible exactly by considering the key postulates of a syntactic theory, and by decomposing them or reverse engineering them to arrive at the earliest stage of grammar (e.g. (10, 34). That was the crucial step that led to the identification of “living fossils,” that is, constructions in present-day languages that best approximate this type of grammar (for examples, see Evidence for sexual selection from colorful “fossils”).

This reconstruction is based on the syntactic theoretical framework associated with minimalism, adopted widely especially in the United States. As discussed subsequently in this section, this reconstruction also turns out to be compatible, even synergistic, with the findings based on other linguistic frameworks. According to minimalism and its predecessors, a modern clause/sentence is highly hierarchical, characterized by the following partial hierarchy of syntactic layers [e.g. (35–40); even though I have attempted to make this section as accessible as possible, it still contains some technical details that pertain to linguistic theories. But this section can be skipped, and the reader can proceed to Evidence for sexual selection from colorful “fossils”]:

**Table:** 

(1)	CP > TP > vP > VP/SC

The inner VP (verb phrase)/SC (small clause) layer typically accommodates one verb/predicate and one argument (noun). The little vP layer is a second layer of a verb phrase, which supports transitivity by accommodating an additional argument (noun), such as agent. The TP (tense phrase, or sentence) layer accommodates the expression of, for example, tense and subjecthood. The complementizer phrase (CP) is yet another layer of structure postulated in this syntactic framework. It facilitates sentential embedding, as well as question formation. The CP will not be the focus of this article (for some discussion of CP in the context of language evolution, see Progovac (10). The theoretical construct in example 1, one of the most stable postulates of this framework, offers a precise and straightforward method of (internally) reconstructing the initial syntactic stage(s) in evolution, by relying on reverse engineering (10, 34). While the SC/VP is composed first as the bottom layer, and it can be composed without a little vP or TP layers, the vP and TP layers, on the other hand, can only be built upon the foundation of an SC/VP. One can thus reconstruct a vP-less and TP-less (intransitive and tenseless) freestanding small clause stage in the evolution of language, consisting of just one verb and one noun. The use of the term *reconstruction* here is based on the method of reverse engineering, but it is also consistent with the characterization of “internal reconstruction” in historical linguistics, considered to be “an extremely useful and generally quite accurate tool for the reconstruction of linguistic prehistory” [e.g. (41)]. It is based on the assumption that languages reveal evidence of past changes in their present structures, and that certain kinds of present alternation in a language can be reconstructed back to an earlier stage in which there was no alternation of that kind [e.g. (42, 43)]. In the context of this article, as will be illustrated in the following sections, this concept is applied to the alternation in the structure of compounds and sentences, both of which have more basic (“fossil”) counterparts. As will be seen subsequently, this internal reconstruction is also reinforced by typological considerations of the relevant syntactic differences across language. This kind of (proto)grammar cannot distinguish subjects from objects, as discussed further in Evidence for sexual selection from colorful “fossils.” The following sections introduce the approximations or “fossils” of this stage, as well as hypotheses and experiments that address their relevance for language evolution.

An advantage of using a precise linguistic framework to reconstruct the linguistic past is that it avoids impressionistic claims, and that it can provide enough specific detail to allow for testing, including neuroimaging experiments (Evidence from neuroimaging). By using this specific syntactic framework, I do not intend to imply that this framework is the only one available, or the best available, but only that it provides some especially useful postulates and tools for a reconstruction based on reverse engineering. While Noam Chomsky and his coauthors on language evolution [e.g. (2, 3)] have promoted a saltationist view of the evolution of syntax, based on minimalism, many others have argued against this stance, including some working within minimalism. As also outlined in Clark (44), the linguistic framework by itself does not dictate if one will adopt a gradualist or saltationist stance. The most stable postulates of this framework, introduced in the previous text, are fully compatible with a gradualist approach. On the other hand, some most recent postulates which seem to support a saltationist view, such as the strong minimalist thesis, are also highly problematic from the theoretical point of view, including being unfalsifiable (see, for example, a review of this work in Progovac (45)]. In this respect, it is rather encouraging that several different linguistic frameworks in fact prove to be compatible with, or even synergistic with, the proposal outlined here. First, the idea of syntactic living fossils as presented here owes to the work of Ray Jackendoff, an opponent of Minimalism. Jackendoff (46, 47) advocates a gradualist view of language evolution, and he lists a variety of candidates for “living fossils” of the earlier stages, including compounds such as *snow-man* and *scare-crow*, the latter a verb-noun compound of the type discussed in this article (Evidence for sexual selection from colorful “fossils”). These two gradualist approaches, as well as other approaches to language evolution, are compared and contrasted in some detail in Progovac (48).

Adopting yet another, completely different framework for studying language and its evolution, grammaticalization, Heine and Kuteva (43) reconstructed an early stage in language evolution which included only nouns and verbs. This is synergistic with the proposal based on syntactic considerations that the earliest stage of grammar involved just simple combinations of one verb and one noun, foreshadowing sentences in present-day languages, the vast majority of which feature noun-verb combinations at their core. Heine and Kuteva's reconstruction is based on the finding, from an abundance of crosslinguistic historical evidence, that grammaticalization processes tend to work in one direction, that is in the direction of developing more abstract and more grammatical words, from more concrete and more content-full words. More specifically, nouns and verbs are reconstructed as the earliest word categories because other word categories, including adjectives, prepositions, auxiliary verbs, subordinators, etc. can be grammaticalized from (i.e. historically developed from) nouns or verbs but typically not the other way around. In that sense, their reconstruction is also based on reverse engineering. It is thus significant that these two reconstructions, based on completely different linguistic frameworks, are directly synergistic. This finding of synergy is only possible when specific linguistic detail is provided.

Another point of synergy can be found with the linguistic field of typology, specifically concerning cross-linguistic variation in the expression of the higher layers of syntactic structure, as depicted in example 1. While languages of the world share the (small clause) foundation involving verbs and nouns, they can differ significantly in how they express the higher layers, including the vP layer, responsible for expressing transitivity and for differentiating subjects from objects. By analyzing the structure of languages across the globe, typologists have classified languages into 3 basic types: (i) nominative-accusative, (ii) ergative-absolutive, and (iii) serial verb types. As shown subsequently, minimal tinkering with the postulated foundational verb + noun (V + N) composition, by adding just one extra piece, yields exactly the 3 vastly predominant transitivity language types [e.g. (34, 48)]. If one extra piece (N) is added from below (as a patient argument), one gets a nominative-accusative language type, such as English (example 2). If, on the other hand, an extra piece (N) is added from above (as a higher, agent-like argument), one gets an ergative-absolutive language type, such as Tongan (example 3) (49). Finally, serial verb language patterns duplicate the whole NV small clause, stringing together a limited number of such clauses, often just two, as in (4) from Anyi-Sanvi, Niger-Congo (50).

**Table:** 

(2a)	Mary shook.						N_1_V	
(2b)	Mary shook Bill.						N_1_VN_2_	
(3a)	Oku	ui	‘a	Mele.			V N_1_	
	_PRES_	call	_ABS_	Mary				
	‘Mary calls.’/‘Mary is called.’							
(3b)	Oku	ui	‘e	Sione	**‘**a	Mele.	V N_2_ N_1_	
	_PRES_	call	_ERG_	John	_ABS_	Mary		
	‘John calls Mary.’							
(4)	Cùá	c̀i	ákɔ́	^!^ dì			N_1_V_1_	N_2_V_2_
	dog	catch + _HAB_	chicken	eat				
	‘The dog eats a chicken.’ (The dog catches, the chicken gets eaten.)							

Such serial verb constructions have one noun per verb, and the first small clause introduces the agent (or the higher argument), and the second clause introduces the patient (or the lower argument). By varying the verb in the first clause, one can express different aspects of the event. In the example below from Aboh (51), the first verb (collect) is used not only to introduce the agent (Àsíbá), but also to express the abundance of the action.

**Table:** 

Àsíbá	bɛ́	lɛ́sì	ɖù	N_1_V_1_	N_2_V_2_	
	Asiba	collect	rice	eat		(5)
	‘Asiba ate a lot of rice.’					

In this sense, each transitivity type represents a different pathway toward the same goal, the goal of capturing an event by differentiating agents from patients, but the starting point of this pathway is not random. Indeed, the starting point is precisely the common foundation provided by the reconstructed ancestral protogrammar, consisting of a combination of just one noun and one verb. This is consistent with the understanding that evolution's creative forces lie in tinkering with the existing, available structures, by combining, recombining and duplicating their pieces (52, 53). This means that even the very essence of syntax, which is often considered in the theoretical circles to be abstract and beyond the reach of biological forces (2–4), is in fact best understood by invoking such forces. According to Marcus (53), the human mind is a product of “haphazard construction.”

## Evidence for sexual selection from colorful “fossils”

### Reconstructed linguistic “fossils” of early grammars

The significance of reconstructing this kind of early stage of grammar is that we can now identify its approximations or proxies in present-day languages, that is, their “living fossils.” The idea of syntactic “living fossils” in this sense was introduced in Jackendoff (46, 47) in the context of language evolution, and it was adopted and extended to new postulates in Progovac (10); the scientific value of this construct was defended in Progovac (48).

These proxies can now be manipulated in experiments, as well as reveal what kinds of functions and benefits the earliest grammars would have brought about. It is often stated by skeptics that “language leaves no fossils” and that thus the study of language evolution is not feasible. In fact, given this proposal, the fossils of language may be much more accessible than what we typically consider as fossils in the archeological sense. This gradualist approach to language evolution and variation views syntax like a quilt, stitched together from a variety of patterns and structures, accrued at various junctures in language evolution. This is also how geneticists describe the human genome: as “a patchwork quilt … with segments that were picked up at different stages of our ancestry” (54). In this sense, human language never really died, but rather just tinkered and tempered with the initial stages. Decomposing human grammars into their evolutionary primitives not only reveals the ancestral compositions, but it can also render them alive in experiments (Evidence from neuroimaging).

There are several types of approximations of the earliest stage postulated for language evolution, and they can be found in different forms and guises across different languages (10, 48), but the ones most closely approximating the reconstructed earliest grammars, and the ones most directly interacting and intersecting with biological considerations, are verb-noun compounds, as illustrated in this section. The examples from English (example 6) are mostly taken from Weekley (55) and the Serbian examples (example 7) are mostly from Mihajlović (56). It should be pointed out that this compounding strategy can be productive (more modern) in some languages (e.g. Romance), acquiring a more complex form, where they would not be considered as “fossils” in the same sense, as discussed in detail in Progovac (48). In both languages, this strategy for forming compounds is no longer fully productive, and has been largely replaced by the more complex, more compositional -*er* strategy, which now clearly identifies the noun as an object of the verb (e.g. *joy-kill-er*, *whistle-blow-er*, *truck-drive-er*). Yet another reason to treat these verb-noun compounds as “fossils” is the fact that linguists typically consider them to be somehow exceptional, or “exocentric,” that is, without a center or a head, defying the rules of modern syntax. Thus, a turncoat is not a kind of coat and a pickpocket is not a kind of pocket. This is directly related to the inability of this kind of two-slot grammar to distinguish subjects from objects, as discussed in detail in Progovac (10, 48).

**Table:** 

(6)	kill-joy, turn-skin/turn-coat (traitor), hunch-back, wag-tail, tattle-tale, scatter-brain, cut-throat, mar-wood (bad carpenter), busy-body, cry-baby, break-back, catch-fly (plant), cut-finger (plant), tumble-weed, fill-belly (glutton), lick-spit, pinch-back (miser), shuffle-wing (bird), skin-flint (miser), spit-fire, swish-tail (bird), rattle-snake, stink-bug, tangle-foot (whiskey), tumble-dung (insect), crake-bone (crack-bone), shave-tail (shove-tail), fuck-ass, fuck-head, shit-ass, shit-head
(7)	ispi-čutura (drink.up-flask—drunkard); guli-koža (peel-skin—who rips you off); cepi-dlaka (split-hair—who splits hairs); muti-voda (muddy-water—trouble-maker); vrti-guz (spin-butt—fidget); tuži-baba (whine-old.woman; tattletale); pali-drvce (ignite-stick, matches); jedi-vek [eat-life = one who constantly annoys]; kosi-noga [skew-leg = person who limps]; mami-para [lure-money = what lures you to spend money]; pali-kuća [burn-house = one who burns houses]; podvi-rep [fold-tail = someone who is crestfallen]; priši-petlja [sow-loop = who clings onto another]; probi-svet [break-world = wanderer]; raspi-kuća (waste-house = who spends away property]; vuci-batina [pull-whip = good-for-nothing]; kaži-prst (say-finger = index finger); jebi-vetar (fuck-wind—charlatan); češi-guz “scratch-butt;” deri-muda “rip-balls” (place name, a steep hill); gladi-kur “stroke-dick” (womanizer); kapi-kur “drip-dick” (name of a slow water spring); liz-guz “lick-butt;” nabi-guz “shove-butt;” piš-kur “piss-dick;” plači-guz “cry-butt;” poj-kurić “sing-dick” (womanizer); seri-vuk “shit-wolf’

These compounds demonstrate the expressive power of just a single merge of two words, a verb and a noun (vs. just using isolated words). Relying on the simplest type of grammar featuring the building blocks of modern languages, that is, nouns and verbs, these compositions are nonetheless highly expressive and versatile. Importantly, once these kinds of rudimentary grammatical compositions became entrenched in populations, they would have provided an excellent scaffolding for evolving more complex grammars, layer by layer (Evidence from linguistic theories: Precise reconstruction of the earliest grammars). Evidence from linguistic theories: Precise reconstruction of the earliest grammars also explains how this small clause foundation provides a common denominator, that is, a common foundation for building typologically different transitive patterns across language. They are rich in colorful metaphors and humor, and they turn out to be especially useful for naming and nicknaming, often in derogatory, playful terms. There were thousands of such verb-noun compounds reported in medieval times, certainly more than nature needs; however, such creative compositions do not get preserved in dictionaries or grammar books because they often show “unquotable coarseness” (55). They tend to have transient lives, with many of them now obsolete, and with different generations being familiar with different ones.

There are some clear generalizations to be made regarding these verb-noun compounds, once one accumulates a critical mass of such data [for many more examples and details, see Progovac (10)]. The verbs and nouns used in these verb-noun compounds tend to have concrete reference, but their combinations can describe rather abstract concepts, often exhibiting stunning feats of metaphorical creativity. Grammatically speaking, the noun in these compounds is not specified as either subject-like or object-like. To illustrate with just one example, while English *tumble-weed* is a weed that tumbles (*weed* is subject-like), *tumble-dung* is an insect that tumbles dung (*dung* is object-like), rendering these compounds as rather close approximations of the reconstructed *intransitive* small clause stage in the evolution of grammar (Evidence from linguistic theories: Precise reconstruction of the earliest grammars).

Such compositions are also found in non-Indo-European languages, such as Berber (example 8) and Twi (example 9). Not only that, but these compounds reveal some striking similarities in imagery across languages and continents (example 10), demonstrating quite similar ways in which humans broke into the metaphorical realm by combining words, enabling them to gradually and radically enrich their vocabularies and grammars, but also their cognition.

**Table:** 

(8)	Tashelhit Berber (spoken in Morocco; Dris Soulaimani, p.c., 2007)	
	slm-aggrn	[suck.in-flour, butterfly]
	ssum-izi	[suck-fly, thrifty person]
	ssum-sitan	[suck-cow, insect] (cf. *burst-cow* in Old English)
(9)	Twi (spoken in Ghana; Kingsley Okai, p.c., 2011)	
	atoto-botom	[dip-in pocket, pickpocket]
	kukru-bin	[roll feces, beetle]
	nom-mmogya	[suck blood, vampire]
	wodi-nii	[kill person, killer]
(10)	tumble-dung (beetle in English) (cf. the equivalent in Twi above, *kukru-bin*)	
	burst-cow (insect in Old English) (cf. Tashelhit *ssum-sitan* above)	
	stink-bug in English (cf. *smrdi-vrana* [stink-crow] in Serbian)	
	wag-tail (bird in English) (cf. *verti-hvostka* in Russian)	
	pica-flor (peck-flower, humming bird in French) (cf. *kjuj-drvo* [peck-wood,	
	woodpecker] in Serbian)	

The importance of identifying proxies of early language in human evolution cannot be overestimated, as the detail they provide is our best bet at advancing testable hypotheses regarding human evolution as it relates to language. These proxies not only provide a fertile ground for experimental testing (Evidence from neuroimaging), but they also allow us to draw insights into how such compositions were used in the earliest stages of language, and thus to advance scenarios for such evolution, including the sexual selection dimension (next subsection).

These proxies also help reveal where continuity with other species should be sought [for some details, see Progovac (57)]. Other primates are in principle capable not only of rudimentary two-slot combinations, but also of playfulness and humor. For example, bonobo Kanzi was reported to use verb-noun combinations such as *hide peanut* and *hide Kanzi* (58). According to Patterson and Gordon (59), gorilla Koko was capable of producing not only novel metaphorical compounds (e.g. “cookie rock” for a stale bun), but also insult, playfulness, and humor. Playfulness and humor that do not rely on words and word combinations are certainly attested in other species, and in humans, too, including children, and they should be seen as precursors and a foundation for wittiness, in which wittiness can be seen as superimposing language upon this foundation.

The earliest form of humor in young children, incongruity-based humor, relies on principles of discrepancy applied to, for example, actions, such as clowning and acting silly (60). As discussed in Grammatical basis of metaphors and humor: The role of sexual selection, Initiation of humor, including clowning, is considered by, for example, McGhee (60) to reveal strong assertiveness, which is again relevant for sexual selection. Language-based humor associated with wittiness also relies on incongruity, but this time on the incongruity coming from words and word combinations, triggering metaphorical extension. According to the Merriam-Webster Dictionary, wittiness is defined specifically with reference to language, in fact metaphorical language: “Using figurative language allows a writer to be both playful and to communicate information effectively to readers. It provides tools to *paint a picture* with words (the words are bringing images to the reader's mind).” This brings metaphors and humor together, and it also highlights the artistic dimension of language, in the sense that the skillful combinations of words can “paint a picture.” This artistic quality is in addition to the potentially pleasing quality of sounds, including the creative use of sounds and intonation. Those who are considered as good speakers often use ornaments in their speech, including adjusting the volume and rhythm of their speech. To take just one example, in Central Brazil the “plaza speech” of Suya Indians has a special style of delivery in which “the phonetic and rhythmic features of speech are altered, generally by exaggerating rhythms and stressing unstressed syllables” (61).

### Evidence for sexual selection: Survival of the wittiest

As established previously, these compounds specialize for insult/derogatory reference when referring to humans, and are often obscene/vulgar, depicting body functions and/or body parts. In addition to many other useful functions, the ability to coin such colorful compounds would have constituted a highly adaptive way to compete for status and sex in ancient times, by, for example, attaching names or nicknames to people (9, 10, 34). The successful use of such two-slot compositions would have enhanced relative status first by derogating existing rivals and placing prospective rivals on notice (intrasexual selection), and second by demonstrating verbal skills and quick-wittedness to the opposite sex (intersexual selection). These are exactly the two types of sexual selection scenarios identified as early as in Darwin (1) [see also Miller (6) and Seeger (62)]. Regarding intrasexual selection, that is, male-on-male competition, the compounds introduced previously often describe men in derogatory terms, but even when they seemingly describe women (e.g. *lezi-baba* “lie-old-woman,” meaning roughly “loose woman or man’), such compounds are still typically used to derogate men, for a doubly insulting effect (9, 56). According to, for example, Marsh (63), the most common type of insult among men even in present times is the kind meant to emasculate the opponent. Both types of sexual selection proved to be relevant in the experiment by Franks and Rigby (7), which found that males increase their creativity with language both in the presence of attractive females and in the presence of male competitors.

In addition to positive selection due to superior language skills and eloquence, it is also important to acknowledge the role of negative selection with respect to these skills, as even minor language disturbances or “disorders” can have a detrimental effect on selection. Especially damning seem to be insults that directly refer to such skills, such as *dim-witted*, *half-witted*, *fuckwit*, *slow*, and *dull*, indicating the value that is still placed on wit and quick-wittedness. This suggests that quick-wittedness plays a role both in positive and negative selection in humans, even today. As discussed in Humor, humor itself is also subject to sexual selection, demonstrating sexual dimorphism.

It would have constituted an unprecedented cognitive leap to become fluent in this strategy of combining, on the spot, two very basic, concrete words in order to express a completely novel, abstract concept. Not everybody was good at it at the time, perhaps only a few were, and those would have been the ones to pass on their genetic make-up through many generations. This initial stage of protogrammar would have unmasked, and thus opened for selection, these otherwise latent cognitive abilities of some of our ancestors. As pointed out in the previous subsection, primates are in principle capable not only of rudimentary two-slot combinations, but also of playful insult. For this to have worked, the witty in these early stages needed to be so expressive, so creative with what little language and grammar there was at that time to be able to outdo the eons-deep exchanges and displays of physical strength. They also needed to have had a very appreciative, discerning, receptive audience. The best way to characterize this scenario is as “the survival of the wittiest.”

## Evidence from neuroimaging

### Verb-noun compounds and the fusiform gyrus: The role of naming

In a functional magnetic resonance imaging (fMRI) experiment, Progovac et al. (64) contrasted the processing of the “fossil” verb-noun compounds (e.g. *kill-joy*; *pick-pocket*; *cry-baby*) vs. the more hierarchical counterparts (e.g. *joy-kill-er*; *boot-lick-er*; *whistle-blow-er*). They found that the processing of flat(ter) (fossil) verb-noun compounds implicated the inferior temporal gyrus and the fusiform gyrus, specifically the right Brodmann's area (BA) 37. BA 37 is the area where visual processing and noncompositional semantic processing (e.g. concreteness, metaphor) come together [e.g. Bookheimer (65)]. According to Ramachandran and Hubbard (66), the beginning of cross-modality involved a cross-wiring in fusiform gyrus. Cross-modality is crucial for processing metaphors, upon which human language heavily relies. Verb-noun compounds evoked a more vivid, more visceral effect, arguably due to the simpler, ancestral grammar, which allows the processing of vivid, expressive language more directly, without the need to also process abstract grammatical words and categories.

These areas, in particular BA 37, are also involved in face perception and recognition (67–70). In fact, as pointed out in, for example, Weibert and Andrews (71), face recognition seems to be facilitated primarily by the right (as opposed to left) BA 37, the area that was also implicated in the processing of verb-noun compounds in the experiment introduced previously. Recall that these compounds are typically used for naming/nicknaming purposes. Crucially, when these simple verb-noun protogrammars started to be entrenched, they would have supported a variety of functions, including naming people in imageable, playful, often pejorative ways. In fact, this was probably one of the earliest successful strategies for naming. These compositions can depict a person based on what they do, how they move and behave, and how they look (e.g. Serbian skew-leg (person who limps); spin-butt/spin-tail (fidget); English *fill-belly* (glutton); *hunch-back*; *scatter-brain*; *cry-baby*; *kill-joy*). In fact, the finding that the fusiform gyrus area in humans is involved not only in both face recognition and naming, but also in the processing of “fossil” grammars, opens up an intriguing possibility that the enhanced face recognition in humans was facilitated by, or coevolved with, the early emergence of language, specifically the early protogrammatical means of naming people, which is essentially an act of associating linguistic labels with people's faces (72).

This ancestral protogrammatical strategy, at least what is preserved of it in modern languages, does seem to be particularly fit for naming, as reported in various previous references. According to Weekley's (55) book titled *Surnames*, devoted specifically to these compounds, there were thousands of such verb-noun compounds created in medieval times, constituting an “expressive way of *naming*.” For Darmesteter (73), the artistic beauty and richness of these compounds (in French) is inexhaustible: “it would be well could French poets again make use in lofty poetry of this class of *epithets*, for they may attain Homeric breadth…” Mihajlović (56), who devoted his career to collecting over 500 Serbian people and place names in the form of these verb-noun compounds, calls them condensed compositions that pack in them frozen fairy tales, proverbs, and ancient wisdoms and metaphors. These reports are consistent with the proposal that there is an artistic quality to these compounds.

In other words, the examples of these verb-noun proxies preserved across languages are primarily used for naming, and they tend to be highly expressive and metaphorical, as well as (playfully) derogatory when referring to humans, thus bringing together both the artistic dimension of language and (verbal) aggression, as further discussed in Evidence from genetics: What do grammar, taming of aggression, humor, and metaphoricity have in common? They would have provided our ancestors with a memorable naming strategy. Such compositions make use of meager means (basic, crude vocabulary and rudimentary syntax) to express highly abstract, novel concepts, which is exactly what would have been needed at the earliest stages of language, ultimately contributing to creating a species that values cognitive contest much more than physical fighting.

### Small clauses and Broca's-basal ganglia networks: Processing of syntax

In the same fMRI study, Progovac et al. (64) tested how flatter (fossil-like) small clauses (e.g. *Problem solved*; *Crisis averted*; *Obama elected*) are processed by the brain, in contrast to their more hierarchical counterparts (i.e. full sentences: *The problem was solved*; *The crisis was averted*; *Obama was elected*). The latter, but not the former, feature a full expression of the syntactic postulates referred to as TPs and determiner phrases (DPs) (Evidence from linguistic theories: precise reconstruction of the earliest grammars). The study found that the “fossil” small clauses resulted in reduced activation both in the left Broca's area and the right basal ganglia, confirming the relevance of cortico-striatal brain networks for the processing of incrementally more complex syntax. In a related example experiment with Serbian participants, Progovac et al. (74) also found the relevance of cortico-striatal networks, most notably the role of basal ganglia, for the processing of the so-called middles vs. fully transitive sentences with vP in Serbian. These experiments provide proof of concept that syntax can not only be decomposed into evolutionary primitives, but that the processing of such primitives can be tested experimentally across different languages and constructions.

Various previous studies on the processing of syntax have converged on the conclusion that it largely depends on a large circuit that involves different cortical areas including Broca's area, but also subcortical structures, including the striatum in the basal ganglia (e.g. (64, 74–83). Importantly for the arguments in this article, the connectivity of the Broca–basal ganglia network has been enhanced relatively recently in evolution, in the line of descent of humans (and Neanderthals), implicating *FOXP2* and other genes (see, for example, (84–86). These findings, bringing together linguistic and genetic considerations, are compatible with the view that the pressures to master certain language skills were at least partly responsible for driving the selection processes which increased the connectivity of the Broca's-basal ganglia network. At the very least, these experiments show that the postulated fossil structures are processed differently by the brain, providing fertile ground for further hypothesizing and testing. The genetic dimension is further considered in Evidence from genetics: What do grammar, taming of aggression, humor, and metaphoricity have in common?

## Grammatical basis of metaphors and humor: The role of sexual selection

### Metaphors rely on grammar

While one can certainly identify precursors to wittiness in other species and in non-verbal domains, including playfulness (Reconstructed linguistic “fossils” of early grammars), still, you cannot be very witty with just single, isolated words. Crucially, it is only in a two-word stage, when words started to be combined, that metaphorical expressions could take off in a rather significant way, magnifying to a large extent the potential for humor. Extrapolating from simple observations about one's surroundings (11), the meaning could now be stretched to create novel concepts and abstract ideas simply by combining words in unexpected, incongruous ways, as shown with short sentences in (12) and with verb-noun compounds in (13).

**Table:** 

(11)	Eagles fly. Leaves fly. Eagles soar. Eagles sink. Babies cry.
(12)	Heart sinks. Heart soars. Time flies.
(13)	scatter-brain, split-hair, muddy-water, cry-baby, spin-butt (fidget), kill-joy

Metaphors are born of pragmatically incongruent *combinations* and rely on the resolution of such incongruity (87), which is also the case with humor, as discussed below. The key to this process is composition or combination, that is, grammar, as incongruity arises exactly in (unexpected) *combinations*. The major milestone in human evolution, both linguistic and cognitive, would have been exactly the emergence of this kind of rudimentary grammar, that is, a stage when words started to be combined into meaningful units, and when unexpected combinations could take our ancestors into the realm of abstraction and imagination. In fact, human language in everyday life is so saturated with metaphors, some old and some new, that we are not even aware of the vast majority of them [see, for example, (88)].

In order to be witty and funny it certainly helps to have some words and some grammar, as discussed above. But, as illustrated with the compounds introduced in the previous sections, not much grammar is needed at all. In fact, the simplest of syntax/grammar, and the earliest of stages, would have sufficed to get this process off the ground. Coining such metaphorical verb-noun combinations at the point when words were few and the grammar rudimentary certainly required novelty, imagination, and quick-wittedness. If humans still appreciate the beauty and creativity of linguistic expression, it stands to reason that this would have also been the case with our ancestors, probably to a much higher degree, as the novelty at that point in evolution was immense.

Metaphors are a product of a creative mind, but also of a grammatical mind, the mind capable of combining words, and these two aspects are inextricably linked in human evolution and in human cognition, as further elaborated in Evidence from genetics: What do grammar, taming of aggression, humor, and metaphoricity have in common?” While Lakoff and Johnson (88) concluded that metaphors are essential tools in everyday lives, it can also be concluded that metaphors, supported by simple grammars, were essential drivers of human cognitive evolution.

### Humor

In addition to being metaphorical, the compounds illustrated in Evidence for sexual selection from colorful “fossils” are also typically humorous/playful, invoking concrete body images or functions to describe and name people, typically in derogatory terms (e.g. *scatter-brain*; *cry-baby*, *pinch-back* for a miser; *spin-butt* for a restless person; *rip-balls* for a steep hill). Just as metaphorical stretching in word combinations arises due to pragmatic incongruity (recall: *Heart soars*; *Heart sinks*), humor also relies on unexpected turns. According to Vrticka et al. (23), humor engages a core network of cortical and subcortical structures, including temporo-occipito-parietal areas involved in detecting and resolving incongruity, that is, in detecting mismatches between expected and presented stimuli [for discussion and a variety of views, see also (89, 90)]. In this sense, these early and rudimentary verb-noun combinations would have unleashed a potential for both metaphorical expression and humor, the crucial and intertwined ingredients of quick-wittedness.

Humor itself is subject to sexual selection, exhibiting in fact an important characteristic of sexual selection: sexual dimorphism [Nicholson (91) reported an analysis of 3,745 personal ads that found that women sought a mate that can make them laugh, twice as often as they offered to return the favor, see Provine (92)]. As pointed out in, for example, McGhee (60), initiation of humor reveals strong assertiveness, especially given that it involves a risk of failure, that is, jokes “falling flat [as mentioned in Reconstructed linguistic “fossils” of early grammars, the incongruity-based humor is the earliest form of humor in young children, based on discrepancy applied to actions, such as clowning and acting silly, which tends to be initiated more by boys (60)]. Vrticka et al.'s (23) fMRI study found that females, both young and adult, showed a greater activation/emotional reactivity during humor perception than did males. Bressler et al. (93) found that “women preferred those who produced humor for all types of relationships, whereas men preferred those who were receptive to their own humor” [see also (94)]. Bressler et al.’s results suggest that “sexual selection may have operated on men's and women's preferences … in dramatically different ways,” strongly suggesting sexual dimorphism.

It is important to point out that humor is not just an inconsequential decoration on the cake of creative language. According to Vrticka et al. (23), humor serves as a natural stress antagonist by decreasing cortisol levels. Humor has a clear and tangible effect on the body: it affects cortisol levels and provides relief from stress and tension, often leading to laughter and mirth [e.g. (95–98)]. Humor often serves to relax and disarm the opposite sex, but also the competitors, which is directly relevant for sexual selection. The significance of these findings will be taken up in the following section, in which these considerations are cross-fertilized with some biological/genetic aspects of human evolution. There is certainly a dark side to humor as well, given that humor can be hostile, subjecting people to ridicule. This is consistent with acknowledging the complexity of human evolution and human nature, which cannot be reduced to just one dimension, such as friendliness. Humor is both about competition and about cooperation.

## Evidence from genetics: What do grammar, taming of aggression, humor, and metaphoricity have in common?

As pointed out in Evidence from neuroimaging, the processing of complex syntax requires dense connectivity in the cortico-striatal networks, which is a relatively recent evolutionary development with a genetic signature. More precisely, the connectivity of the Broca–basal ganglia network has been bolstered in the line of descent of humans (and Neanderthals), implicating *FOXP2* and other genes [see, for example, (84–86)]. This is consistent with the view that selection specifically for language skills played a role in the evolution of the human brain (see also Evidence from neuroimaging). Contributing further to this argument, metaphoricity, humor appreciation, and taming of physical aggression all rely on these same networks. It is notable that all these dimensions are also clearly evident in the nature and use of the linguistic “fossils” introduced in the previous sections, providing further plausibility for their relevance for language evolution. These protogrammatical compositions are not only humorous and imageable, but also tend to involve insult (i.e. verbal aggression) when referring to humans (9). It seems that we are still in a protolinguistic mindset/mode when using this kind of imageable, derogatory language, which in fact prefers to occur in protogrammatical frames [for reasons and findings in this respect, see Progovac et al.'s (64) fMRI study].

Paradoxically, perhaps, it is exactly in this respect that the proposal of the survival-of-the-wittiest can be cross-fertilized with the survival-of-the-friendliest approach, the latter advocated in, for example, Hare (13) and Hare and Woods (14). As proposed in Progovac and Benítez-Burraco (21), the gradual emergence of verbal means of competition/aggression (which certainly includes wittiness) has been engaged in a feedback loop with the genetic forces targeting the reduction in physical aggression, the latter often associated with the HSD hypothesis [see also Benítez-Burraco and Progovac (99)]. The HSD hypothesis is based on the existence in our species of many of the features found in domesticated animals [see, for example, (100–105)]. Such features include prolonged juvenile period, reduced sexual dimorphism, reduced reactive aggression, reduced response (of the hypothalamic-pituitary-adrenal axis) to stress, and decreased cortisol levels. Most of these features are directly relevant for the considerations of language evolution as proposed in this article. By affording a more adaptive (less lethal) way to compete for status and sex (9), these early items of verbal competition would have reinforced and accelerated the effects of self-domestication, associated with the changes in the human brain, leading to a gradual replacement of reactive physical aggression with verbal and cognitive competition. The emergence and use of such early grammars was an essential, crucial part of this feedback loop, both causing and being caused by the gradual evolution of the neuronal density in the specific networks in the human brain.

This development would have also provided a means for reducing cortisol levels through the use of verbal humor, an important player in the self-domestication hypothesis of human evolution (footnote below). Needless to say, cortisol levels, reactivity and aggressive impulses in humans all have a genetic signature. These considerations provide a fertile ground for identifying and testing a variety of hypotheses about human evolution, some of which were introduced in Evidence from neuroimaging. The testing grounds also certainly include considerations of individual variability in human cognition, ranging across a variety of cognitive conditions, such as autism, schizophrenia, and synesthesia [for an extensive discussion and analysis, see, for example (72, 99)].

## Conclusion

The crucial milestone in human evolution, both linguistic and cognitive, was the emergence and entrenchment of the earliest forms of grammar, which unleashed a host of attributes that will turn out to be uniquely human, including increasingly more complex grammars and vast vocabularies, largely enabled by metaphorical extension, the flair for humor and quick-wittedness, and the means to replace physical aggression with cognitive contest. This proposal identifies specific proxies (or “living fossils”) of such early grammars, which bring together 5 key factors in human evolution: (i) protosyntax (i.e. the earliest stage of grammar), (ii) metaphoricity, (iii) humor, (iv) (verbal) aggression, and (v) naming/nicknaming. These basic ingredients of human evolution are interconnected and intertwined, caught in a feedback loop, in which they both cause and are caused by the gradual evolution of the neuronal density in the specific networks in the human brain, engaging the genetic dimension.

It is here that the continuity with the other species is revealed, both when it comes to the form of the earliest grammars and when it comes to the communicative potential (see Evidence for sexual selection from colorful “fossils”). Still, in addition to revealing continuity, this approach also provides a clear and unambiguous point of departure, a beginning of what will turn out to be uniquely human traits. It assigns an active, causal role to language/grammar in human evolution. Importantly, the level of linguistic detail and the identification of “living fossils” provide a fertile ground for a wide variety of hypotheses to be tested, with a goal of understanding how and why (sexual) selection, specifically for agility with language, has been shaping human nature. The proposal of the “survival of the wittiest” is the only proposal about human evolution that does justice to both continuity and to what is uniquely human, while at the same time capturing both the competitive and the cooperative dimensions of human nature.

We often consider eloquence to be correlated with intelligence, but, regrettably perhaps, being witty cannot be equated with being intelligent or wise, in the sense of making the best decisions, or solving problems in an optimal way. The latter skills are certainly adaptive, but they may be harder to gauge in the context of sexual selection, as they take much longer to evaluate. Quick-wittedness is immediately there to observe and admire. It may seem like a shallow skill, like the colorful, imaginative structures built by bower birds during the mating season (106), but it is a form of art that appeals to some deep aesthetic and emotional aspect of human existence. If we indeed come from a series of generations that selected for the art and beauty of quick-wittedness, then we are genetically predisposed to be attracted to it, frivolous or not.

## Data Availability

There are no data underlying this work.
